# Improvement of Core–Shell Lightweight Aggregate by Modifying the Cement–EPS Interface

**DOI:** 10.3390/ma16072827

**Published:** 2023-04-02

**Authors:** Chaoming Pang, Chunpeng Zhang, Peijuan Li

**Affiliations:** Jiangsu Key Laboratory of Civil Engineering Material, School of Materials Science and Engineering, Southeast University, Nanjing 211189, China

**Keywords:** EPS, lightweight aggregate, hydration products

## Abstract

To improve the interfacial compatibility between cement matrix and expanded polystyrene (EPS) in core–shell lightweight aggregates (CSLA), the effects of sodium silicate, polyvinyl acetate (PVA) emulsion, vinyl acetate–ethylene (VAE) emulsion, acrylic acid, and acetic acid on the cement–EPS interface were investigated. The density of the interface was studied by scanning electron microscopy (SEM), and the effect of interfacial agents on the hydration process of cement was studied by the heat of hydration and induction resistivity. The macroscopic properties of the interface of the CSLA were characterized by the “leak-white” rate, drop resistance, and numerical crushing strength. The results show that the sodium silicate densifies the interface by generating hydration products on the EPS surface. At the same time, organic acid enhances the interfacial properties of EPS and cement by increasing the surface roughness, and allowing hydration products to grow in the surface micropores. In terms of the cement hydration process, both interfacial agents delay the cement hydration. Above all, with comprehensive interface properties, “leak-white” rate, and mechanical properties, VAE emulsion and sodium silicate can achieve the best performance with a final crushing resistance of 5.7 MPa, which had a 46% increase compared with the reference group.

## 1. Introduction

Cement-based materials have good durability, high strength, and fire resistance, which are sufficient to meet the safety standards of buildings, while their inferior thermal insulation properties are insufficient to make buildings energy-efficient.

Expanded polystyrene (EPS) has been extensively used in the construction industry due to its excellent thermal insulation properties, lightweight nature, good vibration absorption properties, low cost, and good chemical resistance [1,2,3]. However, it is restricted by building engineers due to its deficient strength, poor fire resistance, and poor ageing resistance, which affect the safety of the building [4,5]. Thus, different coating schemes or other combined schemes are applied to modify EPS, especially coatings or combinations with cementitious materials. It was found that phenolic resin (PF) coating with fly ash (FA) and aluminum hydroxide (ATH) can significantly improve the fire resistance of EPS. These materials are reported to increase the loss on ignition (LOI) value of EPS foam up to 29%, acquiring the V-0 rating (burning stops within 10 s on a vertical part, allowing for drops of plastic that are not inflamed) [6]. EPS could also be used as partial replacement of the sand in the block mixture for non-loading applications [7]. Furthermore, EPS could also be used as coarse aggregate at the levels of 0%, 25%, 50%, 75%, and 100% by volume. The density of EPS lightweight concrete is about 900–1700 kg/m^3^. The 28 d compressive strengths of EPS concrete range from 12.58 MPa to 23.34 MPa [8]. Larger EPS balls should be used and regularly arranged, and then pouring concrete afterwards. The strength of EPS balls in lightweight concrete decreases by more than 25% relative to concrete that does not contain EPS balls [9].

To reduce the negative impact of EPS on the cement matrix, Pang et al. have used EPS as the core to prepare core–shell lightweight aggregates. It was reported that this kind of aggregate had good crushing strength from 1.6 to 4.5 MPa, and was lightweight, with bulk density from 500 to 705 kg/m^3^. The compressive strength of lightweight concrete with CSLA improved from 7.8 MPa to 8.1 MPa at the density of 1000 kg/m^3^, and improved from 10.4 MPa to 11.8 MPa at the density of 1200 kg/m^3^ [10,11]. However, in practical application, EPS is easy to separate from concrete due to the difference in physical and chemical properties between EPS and concrete, which leads to stratification between EPS and concrete [12]. In addition, this difference also leads to the weak interface transition zone between EPS and concrete [13]. 

The incorporation of hydrophilic polymers can improve the interfacial properties of cementitious materials and EPS materials [14,15]. Most of the current research has used vinyl acetate and styrene butadiene rubber as cementitious material bond modifiers [16,17]. Other modifiers, including ethylene–vinyl acetate (EVA), styrene–butyl acrylate emulsion (SAE), polyacrylic ester (PAE), etc., were also used to improve the interfacial properties [12,15]. According to the research of DA Silva et al., acetate ions caused by EVA hydrolysis reacted with Ca(OH)_2_ to produce hydrated calcium acetate and polyvinyl alcohol, reducing the quantity of Ca(OH)_2_, but, also, EVA-modified cement pastes showed expressive formation of polymeric film deposited on the surface of anhydrous and hydrated cement phases, partially sealing pore walls [18]. VAE can also modify the EPS–cement interface, and increase the thermal properties and mechanical properties of mortars [19]. EPS granules can be hydrophilized by using 0.2% sulfonyl and 0.03% bone glue hydro solution [20]. Chen used triethanolamine and polyvinyl alcohol to modify the surface of EPS, and then wrapped organic cementing composites on the surface of EPS as a “shell”. The results showed that the strength of core–shell lightweight aggregates increased 21% compared with the aggregates without the “shell” [21]. Sodium alginate, xanthan gum, styrene–butadiene rubber emulsion, and diatomite admixtures can also be used in improving the strength and shrinkage behavior of EPS lightweight concrete [22,23]. Above all, the preparation of core–shell structured lightweight aggregates using EPS as the core, along with interfacial modification of EPS using interface agents, would also be a feasible way to prepare better lightweight aggregates.

However, existing studies are based on improving the long-term interfacial bonding effect, and lack research on the characterization of the wrapping effect during the preparation process. During the preparation process, EPS is difficult to be uniformly wrapped by the powder composite during the mixing process. Acid washing was widely used in improving the overall strength of recycled lightweight aggregate concrete because it can wash away some of the adhering materials and make the surface rough [24,25]. EPS has good chemical resistance, but it can also be corrupted by some organic acids [26]. Similarly, EPS modified with organic acids should also be able to improve the roughness and increase its ability to mechanically engage with the cement matrix.

**Figure 1:**
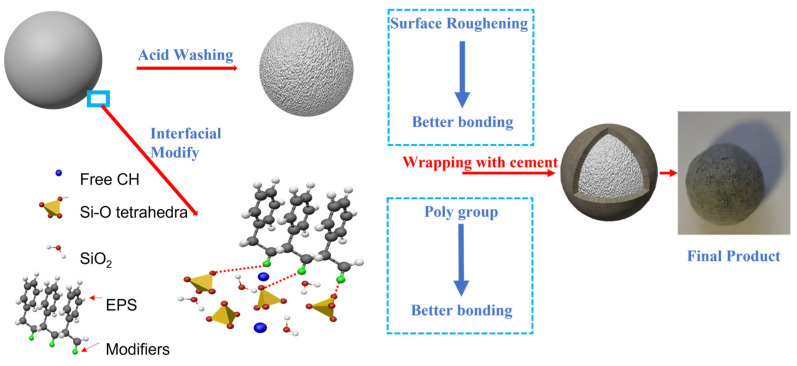
Flow chart of the research.

In this study, as shown in Figure 1, sodium silicate, PVA emulsion, ethylene–vinyl acetate (VAE) emulsion, acrylic acid, and acetic acid were used to modify the EPS surface, in which the first three interfacial agents can enhance the interface adhesion strength mainly by introducing polar groups such as –COOH. In comparison, the last two acidic agents can increase the contact area, and thus enhance the interface adhesion strength mainly by eroding the EPS surface. White EPS rate and falling resistance were tested to ensure the molding properties of core–shell lightweight aggregate (CSLA). Crushing strength and single load-bearing capacity were applied to confirm their achievement in lightweight concrete. Electrical resistivity and heat of hydration were applied to confirm the impact of these interfacial agents on cementitious materials. Then, suitable modification methods were selected to prepare better EPS CSLA by comprehensive analysis of its white EPS rate, drop resistance, microscopic morphology, hydration process, and lightweight aggregate properties.

## 2. Materials and Methods

### 2.1. Materials

PII52.5 cement produced by Jiangnan Xiaoyetian was used in this study and the chemical composition and physical properties was shown in Table 1 and Table 2. The EPS used had a size range of 5–7 mm and a bulk density of 12 kg/m^3^. The sodium silicate was produced by JingCheng Chemical, Shanghai, with a solid content of 51% and modulus of 3.8. Modulus was adjusted to 3, 2.5, 2, and 1.5 by adding a certain amount of sodium hydroxide. The polymer interfacial agents were two different polymer emulsions—PVA emulsion and VAE emulsion. PVA emulsions were produced by Chuanwei Chemical, Sichuan, with a solid content of 20%, and were made by the polymerization of vinyl acetate. VAE emulsions were produced by Boyue Chemical, Henan, with a solid content of 55%, and mainly contained C=O and –OH groups from vinyl acetate and ethylene, which were the raw materials of the polymerization and had good hydrophilicity. The organic chemical reagents of interfacial treatment agents (i.e., acrylic acid and acetic acid) were chemically pure.

### 2.2. Experimental Methods

#### 2.2.1. Aggregate Properties

1. Crushing resistance: The nominal compressive pressure in a cylinder is based on the standard GB/T 17431-2010 “Lightweight Aggregate and its Test Method”. The lightweight aggregate is loaded into a cylinder with an inner diameter of 115 mm and a net height of 145 mm, and a stamping die is used to apply a uniform load at a speed of 300~500 N/s. The pressure value divided by the pressed area is the nominal compressive strength when the stamping die presses in to a depth of 20 mm.

2. Single load-bearing capacity: The single load-bearing capacity of the lightweight aggregate was tested by using a digital display push–pull meter, selecting a fixed loading speed of 10 N/s to press down the aggregate until broken, and recording the peak value.

In the study of molding properties of EPS and cement matrix interface bonding, there was no standard measurement method. However, YB/T 4848-2020, the “physical inspection method of the roasted green ball”, was commonly used in the production process of another spherical material, and a similar experimental protocol was designed. The process of core–shell lightweight aggregate production, due to insufficient interface adhesion strength of the interface when falling from a certain height (1 m, 1.5 m and 2 m), will lead to the bonded powder falling off, and white EPS becoming fully or partially exposed, called the “leak-white” phenomenon. In this study, after using the fall resistance and white EPS rate (WER) after the coating or after the fall to characterize the interface adhesion properties, these two tests can also reflect the core–shell lightweight aggregate rate of finished products’ yield.

3. WER experiment: M pieces of EPS were randomly selected for initial molding with powder, and the “leak-white” phenomenon was observed. The “leak-white” phenomenon was divided into four types: completely “leak-white” (N1), more than 1/2 “leak-white” (N2), less than 1/2 “leak-white” (N3), and without “leak-white” (N4). N1 + N2 + N3 + N4 = 20, where more than 1/2 “leak-white” can be recorded as 75% “leak-white”, and less than 1/2 “leak-white” can be recorded as 25% “leak-white”. Then, the formula for calculating the whitening rate is as follows:

(1) $$WER=\frac{N1+0.75*N2+0.25*N3}{M}$$

4. Falling resistance experiment: M pieces of freshly formed core–shell lightweight aggregates were randomly selected and dropped from a certain height (1 m, 1.5 m, and 2 m), and the number of intact aggregates (no cracks and peeling on the surface of aggregates) after the drop was recorded as N. Then, the formula for calculating the drop integrity rate is as follows:

(2) $$Drop\;Rate=\frac{N}{M}*100\%$$

#### 2.2.2. Morphology of Interface

The CSLA was cut in half, and then one half of the CSLA was soaked in anhydrous ethanol for 3 d, then baked in a vacuum oven at 40 °C to a constant weight. Thereafter, SEM was used to observe the CSLA profile. The experiments were performed using a field emission scanning electron microscope manufactured by FEI, USA, with an accelerating voltage of 200 V~30 kV and secondary electron imaging.

#### 2.2.3. Heat of Hydration

The exothermic rate of hydration of the net cement slurry was measured by a TAM Air thermostatic calorimeter from TA Instruments, New Castle, DE, USA. The mixture (water-cement ratio 0.4) was added to the sample bottle, stirred rapidly and evenly, and immediately put into the thermostatic calorimeter to start the measurement. The temperature was strictly controlled to 20 °C during the test.

#### 2.2.4. Electrical Resistivity

The electrical resistivity was measured by a CCR-2 type dual circuit electrode-free resistivity tester. The mix was prepared according to the ratio (water-cement ratio 0.4), placed in the ring mold of the resistivity tester, and, after excluding the air bubbles of the slurry, the test was started. The data recording frequency was one time/min until the test age.

## 3. Results and Discussion

### 3.1. Effect of Interface Modifier on EPS–Cement Interface

#### 3.1.1. Effect of Different Interface Agents on the Molding Properties of CSLA

**Figure 2:**
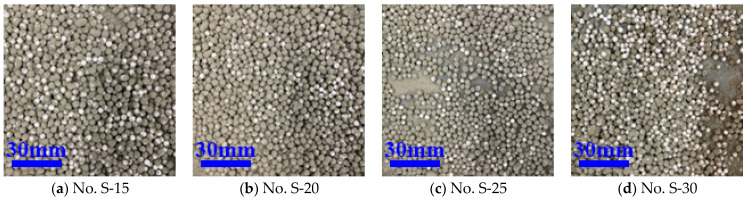
The exposure of CSLA after sodium silicate treatment.

Sodium silicate can improve the adherence of organic materials with cementitious materials, and is a commonly-used early strength agent and interfacial modifier. Also, the modulus of water glass n (Na_2_O·nSiO_2_) has a large effect on properties such as cement setting time and strength [27,28]. Therefore, in this experiment, the water glass solutions with different moduli were selected for interfacial modification of EPS, and the modified treated core–shell light aggregates’ WER were as shown in Table 3 and Figure 2.

**Figure 3:**
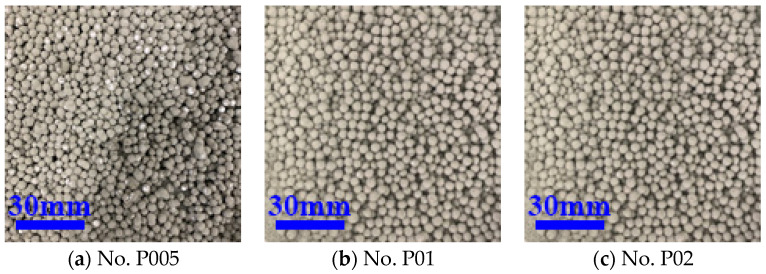
The exposure of CSLA after PVA emulsion treatment.

**Figure 4:**
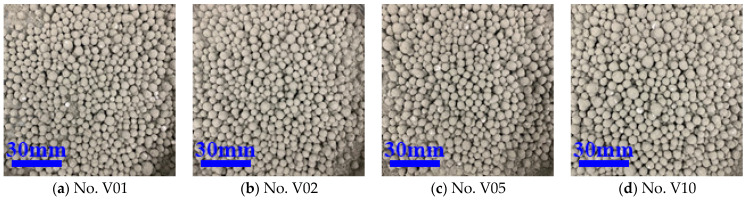
The exposure of CSLA after VAE emulsion treatment.

From Table 3, it can be found that, when the modulus of sodium silicate was in the range of 1.5~3, as modulus increased, the EPS WER first decreased and then increased. When the modulus was 2.5, the core–shell lightweight aggregate WER was the smallest, indicating the best pretreatment effect, as shown in Figure 2c. However, at this time, the WER was still up to 10, because the preparation of core–shell lightweight aggregate was still not small enough, so further use of binder and organic acid type interface agents for interface treatment was proposed. When the modulus of water glass is between 1.5 and 2.5, the number of hydration products increases, the pore structure is refined, and the early strength of cement paste is higher, which enhances the performance of CSLA and reduces the WER; however, as the modulus is further increased, the silica phase gradually increases and shows a deficiency of calcium phases. Thus, the performance of cement matrix decreases instead, leading to a decrease in the performance of CSLA and an increase in WER. The binder polymers PVA emulsion and VAE emulsion were chosen as the interface agents for the experiments because we considered that polymer emulsions can significantly improve the bond strength of the EPS–cement interface, and can improve the toughness and mechanical properties of EPS–cement matrix composites. The WER of the core–shell lightweight aggregate after the binder modification treatment is shown in Table 4, and Figure 3 and Figure 4.

As shown in Table 4, using different concentrations of PVA emulsions from 0.5% to 2% as the interface agent, the “leak-white” phenomenon was better than that of the sodium silicate group before. Additionally, the WER decreased and the interface treatment effect improved as the concentration of PVA emulsion increased, as shown in Figure 4. Because of the poor solubility of PVA emulsion in water, the subsequent use of a 1% concentration of PVA emulsion, as shown in Figure 3b, also possessed a lower WER of 2.5%. Different concentrations of VAE emulsion from 1% to 10% were used as an interfacial agent, and with the increase in the concentration of VAE emulsion, the WER first decreased and then increased. At low dosing, with the increase in the amount of VAE emulsion, the polymer film made the EPS–cement interface bond more tightly, while at higher dosing, the amount of polymer was too much, causing excessive viscosity of mortar, mobility to become small, and an environment not conducive to reducing the WER. The effect of interfacial treatment is shown in Figure 4, and the optimal amount of VAE emulsion is 2%, where WER is 2.5%.

**Figure 5:**
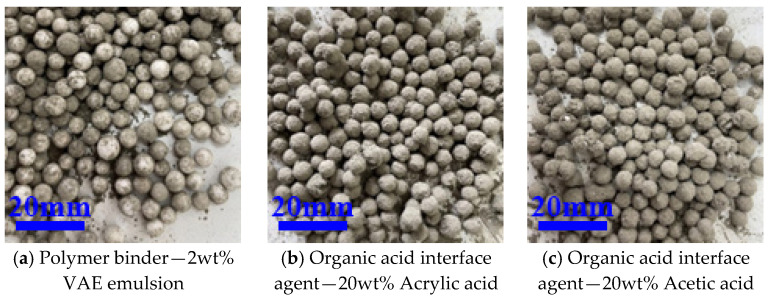
The exposure of CSLA after 1.5 m height falling.

The EPS treated by the binder has a lower WER, but its drop resistance is poor, as shown in Figure 5a, with more “leak-white” phenomenon after dropping, resulting in a lower finished product yield of core–shell lightweight aggregate. The EPS was originally very smooth and had difficulty in bonding cement. If the treated EPS surface has a certain roughness, it is easier for EPS to bond cement, which can improve its drop resistance. Therefore, with the use of acrylic acid and acetic acid as interface agents, the effects of different concentrations of organic acid-based interface agents on the core–shell lightweight aggregate WER are shown in Table 5.

From Table 5, it can be found that the effect of these two interface agents on EPS WER increased as their concentration increased, and the effect of acrylic acid interface treatment is better than that of acetic acid. From the perspective of WER, both acrylic acid and acetic acid concentrations above 20% can achieve a better WER. Compared with the polymer binder, the fall resistance of the aggregates treated by an organic acid interface agent is better. As shown in Figure 5, the shell layer of the aggregates treated by the binder for initial molding was all peeled off after falling, while the aggregates treated by the organic acid interface agent were mostly not peeled off after falling. In the actual production of core–shell lightweight aggregate, the organic acid-based interfacial agent-treated EPS has better fall resistance and is more beneficial for the preparation of core–shell lightweight aggregate.

#### 3.1.2. Effect of Different Interfacial Agents on the Micromorphology of EPS and Cementitious

**Figure 6:**
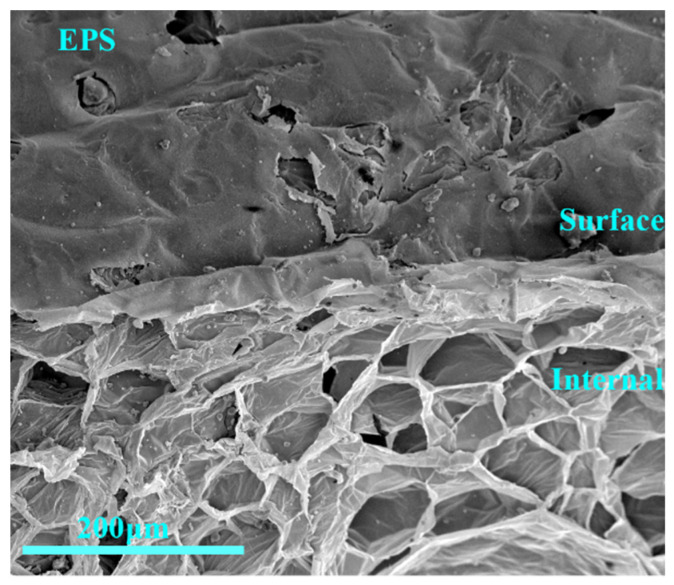
Surface and internal microstructure of untreated EPS.

Untreated EPS surface and internal structure are shown in Figure 6. The internal structure of EPS is porous and laminar, and the surface is covered with a film which is continuous and smooth. EPS–cement interface is not compatible, so the bonding of EPS surface and cement is more difficult. Therefore, different interface agents were selected to treat the EPS surface, and study the improvement of the interface between the cement matrix and EPS after their addition.

**Figure 7:**
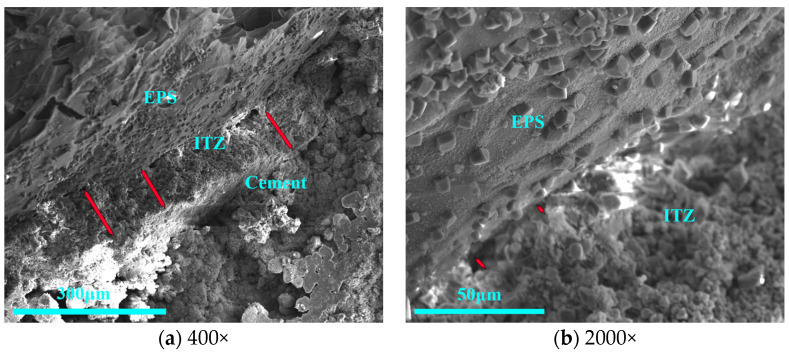
Microstructure of interface treated with sodium silicate.

The EPS–cement interface pretreated with sodium silicate was shown in Figure 7, and it can be found that sodium silicate cannot change the roughness of EPS. There was a dense interfacial transition zone (ITZ) present in the shell layer, and the thickness of ITZ was 83.3~94.2 μm, as shown in Figure 7a. And the interfacial gap was also smaller [29], the interfacial gap was in the range of 2.4 μm to 7.7 μm, as shown in Figure 7b. The sodium silicate, rich in Si–OH bonds, can participate in the hydration reaction of cement, which helps to make an excellent interfacial bond between EPS and cement matrix. Although the EPS–cement interface pretreated with sodium silicate was denser at the microscopic level after hardening, the EPS had a high WER and poor initial interfacial bonding performance, which affects the product’s yield of core–shell lightweight aggregates.

**Figure 8:**
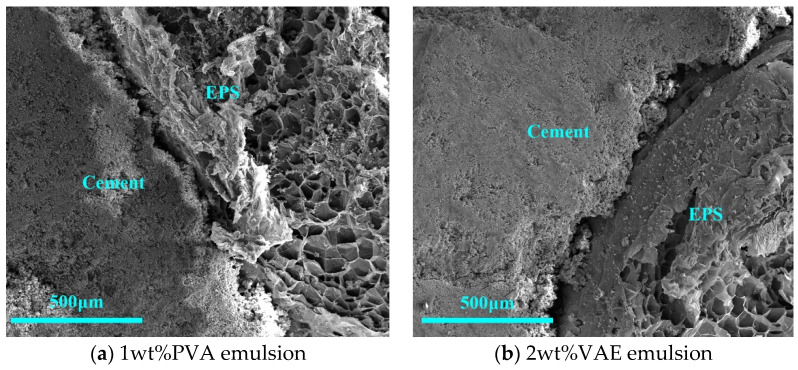
Microstructure of interface treated with the polymer binder.

The effect of polymer binder PVA emulsion and VAE emulsion treatment on EPS–cement interface is shown in Figure 8. Comparing Figure 8 to the sodium silicate group, at the same magnification of 200 times, the two groups of binder in the EPS–cement interface at the gap were larger, and the delamination was obvious, indicating that the polymer binder treatment of EPS–cement microscopic interface was not dense, and, thus, the choice of binder interface treatment alone was not ideal.

**Figure 9:**
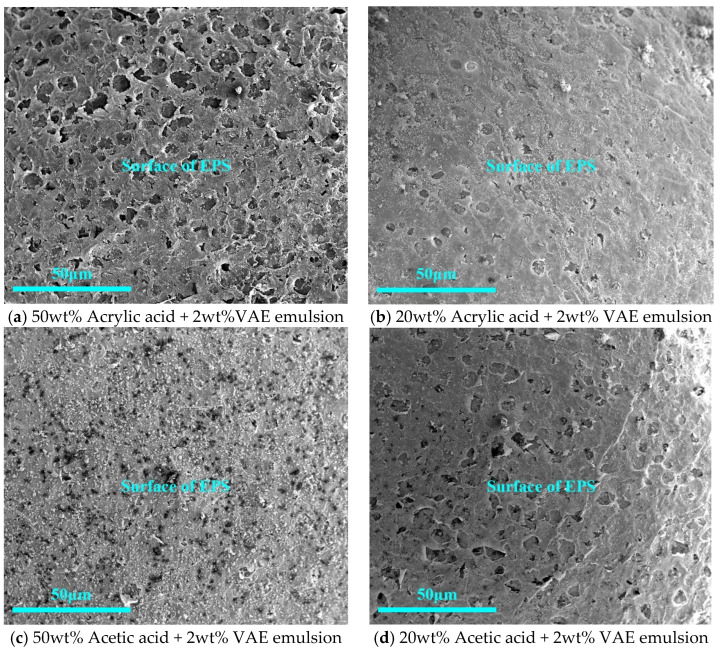
Microstructure of interface treated with an organic acid interface agent.

Based on the previous tests, a class of interfacial agents was considered to increase the roughness of the EPS surface, and the SEM of the EPS after surface treatment with the acrylic acid and acetic acid composite binders is shown in Figure 9. As shown in Figure 9, organic acid interfacial agents—in this work, acrylic acid and acetic acid—can slightly corrode the EPS surface so that the EPS surface becomes rough, with corrosion appearing as relatively uniform holes. With a reduction in the amount of acrylic acid doping, EPS surface corrosion was also reduced. As shown in Figure 9a,d, the corrosion effects of 20% acetic acid and 50% acrylic acid were similar. At the 50% acetic acid interface, as shown in Figure 9c, hydration products firmly covered the EPS surface and could not be peeled. In terms of corrosion effect, the effect of acetic acid is better than that of acrylic acid.

**Figure 10:**
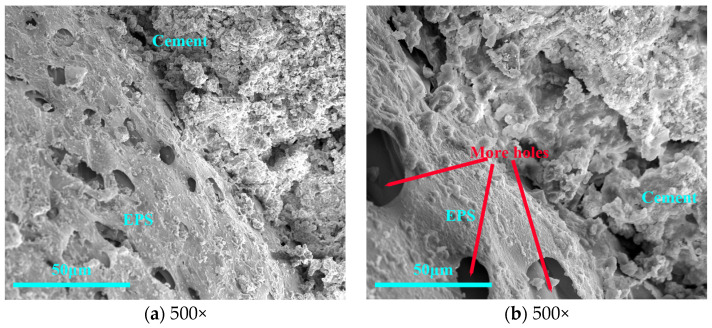
Microstructure of interface treated with organic acid interface agent and VAE emulsion.

Concerning the WER test, the choice of combined acetic acid and VAE emulsion on the EPS surface treatment effect is optimal. The 2% VAE and 20% acetic acid compound treatment EPS–cement interface is shown in Figure 10. Cement hydration products and EPS connection were denser, and, when magnified 500 times in Figure 10b, there was no obvious delamination. The corrosion effect of the organic acid interface agent makes the EPS surface show more holes, and the cement hydration product growth process fills the holes so that the EPS–cement interface becomes dense.

Although sodium silicate can make the interface denser, its WER was still high; in a polymer binder, although the interface was not dense enough, the WER was relatively low. The anti-drop property of both was poor, which was not conducive to the preparation of core–shell lightweight aggregate. The comprehensive molding performance of the organic acid-based interface agent was good, and the compound of acetic acid and VAE emulsion not only had good WER and molding performance, but also had a dense microscopic interface, which met the requirements of the core–shell lightweight aggregate interface agent.

### 3.2. Effect of Different Interface Agents on Cement Hydration

#### 3.2.1. Effect of Single Interface Agent on the Heat of Hydration of Cement

To verify the effect brought by the interfacial modifier on the cement matrix, heat of hydration experiments and resistivity experiments were used to test the hydration process of cementitious materials.

**Figure 11:**
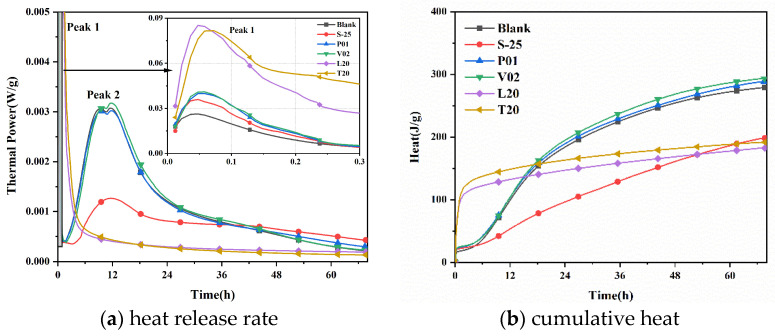
Effect of different interface agents on hydration heat of cement.

Five representative groups of interfacial agents and blank cement groups were selected for the heat of hydration exothermic experiments: S-25 for the sodium silicate group; PVA emulsion P01 and VAE emulsion V02 for the binder group; and organic acid interfacial agents acrylic acid L20 and acetic acid T20. Figure 11 demonstrates the effects of different interfacial agents on the heat of hydration of cement.

As shown in Figure 11, the blank group, sodium silicate group, and binder group all have two exothermic peaks, while the organic acid interfacial agent group only has the initial peak (peak 1). In the initial 5~15 min, the cement is rapidly dissociated when it meets water, and a gel-like film layer is formed on the surface of cement particles, which soon reaches the first exothermic peak (peak 1). The cement then enters the induction period, and the hydration reaction occurs on the outside of the gel film and in the liquid phase of C_3_A, but hydration products adhere to the surface of the particles, hindering the further hydration. At this time, the exothermic peaks of the sodium silicate and binder groups were significantly higher than those of the blank cement group, and the exothermic peaks of the binder group were higher than those of the sodium silicate group. The differences between the PVA emulsion and VAE emulsion groups were not significant, indicating that both the binder and the water glass could promote the dissolution of cement. The organic acid interfacial agent group only had peak 1, and the exothermic peak at this time was more than three times that of the blank cement group, indicating that the organic acid interfacial agent could promote the rapid dissolution of cement in the initial period of hydration.

At the end of the induction period, the acceleration period is entered, and a second exothermic peak is formed. There is no peak 2 in the organic acid interfacial agent group, indicating the lack of an accelerated period of hydration. In the normal hydration process, the accelerated period is mainly characterized by the hydration of C3S, and saturation and precipitation of CH [30], indicating that organic acid-based interfacial agents hinder the hydration of cement C3S and retard the formation of CH. Pan [31] has similar conclusions, and Nguyen [32] also states that the acid even retards the formation of early calcium alumina. The addition of a binder has little effect on peak 2. The addition of 2% VAE emulsion slightly increased the exothermic peak and made the hydration more adequate. However, the addition of sodium silicate with modulus 2.5 significantly decreased the peak and broadened the temperature peak, which slowed down the process of cement hydration because sodium silicate affected the setting time of gel material, and the rate of hydration reaction gradually decreased with the increase of sodium silicate modulus [33].

Figure 11b shows the cumulative heat of hydration of different interfacial agents, and it can be found that the cumulative heat of hydration of different interfacial agents differed significantly. Compared with the blank cement group, the cumulative heat of hydration after adding binder was slightly higher than that of the blank group, and the cumulative heat of hydration of the sodium silicate and organic acid interfacial agent groups were significantly lower than that of the blank cement group. At 72 h, the accumulated heat of hydration of the blank group, binder group, and organic acid type interface agent group tended to be stable, while the water glass group showed an obvious increasing trend.

#### 3.2.2. Effect of Single Interface Agent on Cement Resistivity

**Figure 12:**
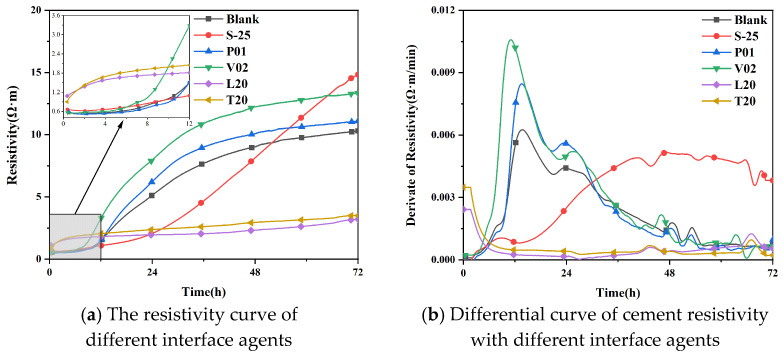
Diagram of the influence of different interface agents on cement resistivity.

The electrical resistivity of cement-based materials changes with the process of cement hydration, so it is usually used to describe the hydration process of cement-based materials and judge the effect of chemical admixtures on cement hydration [34]. The impact of different interface agents on the resistivity of cement are shown in Figure 12.

As shown in Figure 12, it can be found that the addition of polymer binder increased the resistivity compared to the control group. The addition of sodium silicate with modulus 2.5 decreased the resistivity in the first 50 h, and then the resistivity increased significantly after 50 h. The addition of organic acid interfacial agents decreased the resistivity significantly. The differential resistivity curves of different interfacial agents are shown in Figure 12b, and demonstrate the following: the addition of binder accelerated the hydration process, so the peak became larger and also earlier; the addition of sodium silicate significantly reduced the peak and broadened the temperature peak, which slowed down the process of cement hydration; and the addition of organic acid interfacial agent hindered the process of cement hydration.

### 3.3. Effect of Different Interfacial Agents on Core–Shell Lightweight Aggregates

The core–shell lightweight aggregate with an average diameter of 9.4~9.7 mm and a shell layer thickness of 1.8~1.9 mm was prepared by using cement paste as the shell layer material with a w/c ratio of 0.3. The effect of interface agent selection on molding performance and basic performance are shown in Table 6 and Table 7, respectively.

As shown in Table 6 and Table 7, without a suitable interfacial agent, the core–shell lightweight aggregate cannot be prepared by molding, so no blank group was set in this experiment. If only sodium silicate solution were chosen as the interface agent, the prepared core–shell lightweight aggregate would have a relatively high WER. As well, the dew part was not removed in the experiment, which has a more significant impact on the mechanical properties, so the mechanical properties of the S-25 group are poor, and the drop resistance of the core–shell lightweight aggregate was also poor. The performance of core–shell lightweight aggregate obtained by combining binder and sodium silicate was better, such as the P01S-25 and V02S-25 groups, because there was almost no “leak white”, and the interface structure was dense at the microscopic level, so the single-particle load-bearing capacity and numerical tube pressure of core–shell lightweight aggregate were higher. The numerical tube pressure can comprehensively reflect the interface performance of cement and EPS, which is the macroscopic manifestation of the interface bonding performance, so the increase in numerical tube pressure also means the improvement of the interface. Dong [35] also confirms that various emulsions were beneficial to improve the bonding and flexural properties of polymeric mortars. The performance of the core–shell lightweight aggregate prepared with the combination of binder and organic acid-based interfacial agents was better than that of the sodium silicate alone because of the reduced WER of EPS, but the lower numerical tube pressure compared to the combination of binder and water glass group was due to the addition of organic acid-based interfacial agents that affected the hydration process of the cement. From Table 7, it can be found that the compound binder and organic acid interface agent can significantly improve the drop resistance—the 1 m drop integrity rate was increased from 75% to 95~100%—and the improvement of drop resistance can improve the yield of core–shell lightweight aggregate.

The bulk density and crushing strength of other types of cold-bonding lightweight aggregates are shown in Table 8. The crushing strength of the lightweight aggregates in this study was at least 42.5% higher than that of the lightweight aggregates in other studies at similar density classes. Also, Zhou et al. reported that the crushing strength of some fly ash lightweight aggregates can reach 6 MPa, but their bulk density is in the range of 1000–1100 kg/m^3^, which is not suitable for lightweight concrete preparation [36]. In summary, the CSLA prepared in this research solved the contradiction that the common cold-bonding lightweight aggregate with high strength does not meet the density requirements, and the cold-bonding lightweight aggregate which meets the density requirements does not possess high enough strength.

## 4. Conclusions

In this study, WER and falling resistance were applied to confirm the molding properties of CSLA. Crushing strength and single load-bearing capacity were applied to confirm its achievement in lightweight concrete. Electrical resistivity and heat of hydration were applied to confirm the impact of these interfacial agents on cementitious materials. The following conclusions were drawn:

1. With the use of polymer binder as an EPS–cement interface agent, the WER of EPS can be reduced from 10% to 1.25%; the use of an organic acid interface agent can reduce the EPS initial shell WER from 10% to 0%. Pretreatment effect is better.
2. Polymer binder treatment EPS–cement microscopic interface is not dense; an organic acid interface agent can make EPS surface corrosion that is uniformly distributed microporous, and the acetic acid corrosion effect is better than acrylic acid. The 2% VAE emulsion and 20% acetic acid compound EPS–cement interface showed the cement hydration products and EPS surface connection were dense, and magnification of 2000 times proved an EPS–cement interface without obvious delamination phenomenon.
3. Similar patterns can be obtained by using induction resistivity experiments and heat of hydration experiments to study the hydration process. The addition of sodium silicate with modulus 2.5 slows down the process of cement hydration reaction, the binder has little effect on cement hydration, and the organic acid interface agent has a hindering effect on cement hydration.
4. For core–shell lightweight aggregate prepared by different interface agents, WER of sodium silicate-modified EPS is higher, and its mechanical properties are lower; mechanical properties of binder and sodium silicate compound group are better, especially the group of VAE emulsion and sodium silicate compound. The bulk density of about 650 kg/m^3^ core–shell lightweight aggregate have a 28 d numerical tube pressure of up to 5.7 MPa. With organic acid type interface agent added, the initial WER of EPS is lower, EPS cement micro-interfacial properties are better, and the fall resistance is significantly improved, but this affects the hydration process of the cement so that its overall crushing resistance is reduced instead.
5. CSLA in this research did not require a sintering process and had at least a 42.5% increase in the crushing strength compared with other cold-bonding lightweight aggregates, and had at least a 34.5% decrease in bulk density compared with other cold-bonding lightweight aggregates.

## Figures and Tables

**Table 1:** Chemical composition of Portland cement.

Components(wt%)	CaO	Al_2_O_3_	SiO_2_	Fe_2_O_3_	P_2_O_5_	SO_3_	K_2_O	Cr	TiO_2_	Cl^−^	MnO
PII52.5	68.11	4.13	18.17	3.15	0.11	3.79	0.59	0.01	0.19	0.03	0.01

**Table 2:** Physical properties of Portland cement.

	Specific Surface Area	Initial Setting	Final Setting	Compressive Strength	Flexural Strength
PII52.5	338 m^2^/kg	199 min	285 min	53.5 MPa	9.5 MPa

**Table 3:** Effect of sodium silicate with different moduli on the WER of CSLA.

No.	Type of Interfacial Agent	Pretreatment Effect				WER of EPS/%
		Disclosed Quantity	More than 1/2 Quantity	Less than 1/2 Quantity	Undisclosed Quantity	
S-15	Modulus 1.5 of sodium silicate	0	6	9	5	33.75
S-20	Modulus 2 of sodium silicate	0	1	12	7	18.75
S-25	Modulus 2.5 of sodium silicate	0	0	8	12	10.0
S-30	Modulus 3 of sodium silicate	1	8	10	1	40.0

**Table 4:** Effect of organic emulsion on the WER of CSLA.

No.	Type of Interfacial Agent	Pretreatment Effect				WER of EPS/%
		Disclosed Quantity	More than 1/2 Quantity	Less than 1/2 Quantity	Undisclosed Quantity	
P005	0.5% PVA emulsion	0	0	6	15	7.5
P01	1%PVA emulsion	0	0	2	18	2.5
P02	2% PVA emulsion	0	0	1	19	1.25
V01	1%VAE emulsion	1	1	1	17	10.0
V02	2%VAE emulsion	0	0	2	18	2.5
V05	5%VAE emulsion	0	1	1	18	5.0
V10	10%VAE emulsion	0	1	2	18	6.25

**Table 5:** Effect of organic acid on the WER of CSLA.

No.	Type of Interfacial Agent	Pretreatment Effect				WER of EPS/%
		Disclosed Quantity	More than 1/2 Quantity	Less than 1/2 Quantity	Undisclosed Quantity	
L50	50wt% Acrylic acid	0	0	0	20	0
L20	20wt% Acrylic acid	0	0	1	19	1.25
L10	10wt% Acrylic acid	0	1	2	17	6.25
T50	50wt% Acetic acid	0	0	2	18	2.5
T20	20wt% Acetic acid	0	0	3	17	3.75
T10	10wt% Acetic acid	0	1	4	15	7.5

**Table 6:** Type of interfacial agent and forming performance of CSLA.

No.	Type of Interfacial Agent	1 m High Drop Integrity Rate/%	1.5 m High Drop Integrity Rate/%	2 m High Drop Integrity Rate/%
S-25	10wt% sodium silicate solution	75	70	65
P01S25	1wt% PVA emulsion + 10wt% sodium silicate solution	85	85	75
V02S25	2wt% VAE emulsion + 10wt% sodium silicate solution	80	75	75
V02L02	2wt% VAE emulsion + 20wt% Acrylic acid	100	95	85
V02T02	2wt% VAE emulsion + 20wt% Acetic acid	95	95	80

**Table 7:** Basic properties of CSLA.

No.	Bulk Density /(kg/m^3^)	Apparent Density/(kg/m^3^)	Water Absorption/%		Single Load-Bearing Capacity/N			Crushing Resistance/MPa	
			1 h	24 h	3 d	7 d	28 d	7 d	28 d
S-25	612	1245	18.6	19.3	142	160	211	3.1	3.9
P01S25	656	1311	17.1	17.8	221	244	313	4.5	5.5
V02S25	647	1296	16.3	17.4	228	260	334	5.0	5.7
V02L02	641	1289	16.9	18.5	161	186	246	3.8	4.6
V02T02	650	1281	16.8	17.9	177	198	258	3.9	4.9

**Table 8:** Basic properties of other cold-bonding lightweight aggregate.

Raw Materials	Bulk Density/(kg/m^3^)	Crushing Strength/MPa	Literature
73% coal gasification coarse slag, 15% cement, 1% plaster/NaCl/Al_2_(SO_4_)_3_	651	1.00	[37]
Titanium slag, sodium silicate	668	3.67	[38]
71% fly ash, 10% cement, 10% lime, 7% pearlite powder, 2% pore foaming materials	699	3.99	[39]
2~4 mm expanded pearlite, fly ash, cement	500~650	2.0~2.7	[40]
Cement, silica fume	550~608	3.1~3.5	[41]

## Data Availability

Data is contained within the article.
